# Fully Automated AI-Based Lymph Node Measurements in Chest CT: Accuracy and Reproducibility Compared with Multi-Reader Assessment

**DOI:** 10.3390/diagnostics16070967

**Published:** 2026-03-24

**Authors:** Andra-Iza Iuga, Heike Carolus, Liliana Lourenco Caldeira, Jonathan Kottlors, Miriam Rinneburger, Mathilda Weisthoff, Philipp Fervers, Philip Rauen, Florian Fichter, Lukas Goertz, Pia Niederau, Florian Siedek, Carola Heneweer, Carsten Gietzen, Lenhard Pennig, Anja Dobrostal, Fabian Laqua, Piotr Woznicki, David Maintz, Bettina Baessler, Thorsten Persigehl

**Affiliations:** 1 Institute of Diagnostic and Interventional Radiology, Faculty of Medicine and University Hospital Cologne, University of Cologne, 50937 Cologne, Germany; 2 Philips GmbH Innovative Technologies, 22335 Hamburg, Germany; 3 Institute of Neuroradiology, Lucerne Cantonal Hospital, 6000 Lucerne, Switzerland; 4 Department of Radiology, Hospital of the Augustinians, 50678 Cologne, Germany; 5 Department of Diagnostic and Interventional Radiology, University Hospital Würzburg, 97080 Würzburg, Germany

**Keywords:** staging, oncologic imaging, artificial intelligence, AI, CT, computed tomography, lymph nodes, measurement consistency, short-axis diameter, long-axis diameter

## Abstract

**Background/Objectives**: Accurate and reproducible lymph node (LN) measurement is essential for oncologic staging and therapy monitoring but is subject to inter-reader variability. This study evaluated the accuracy and reproducibility of a fully automated artificial intelligence (AI)-based LN measurement workflow in contrast-enhanced chest CT, using multi-reader manual measurements as reference. **Methods**: Sixty thoracic LNs from seven patients were independently measured by 13 radiologists in two reading rounds. The median of all measurements served as the ground truth (GT). Automated short- and long-axis diameters were derived from fully automated 3D CNN-based segmentations. Agreement between AI and manual measurements was assessed using Friedman testing, intraclass correlation coefficients (ICCs), and concordance correlation coefficients (CCCs). Measurement stability was evaluated across repeated runs on different hardware systems. **Results**: A total of 2280 manual measurements were analyzed. Manual assessment showed significant inter-reader variability (*p* < 0.01), while intra-reader agreement was high. No significant differences were observed between AI-based measurements and the GT (all *p* > 0.01). Agreement was good, with CCC values of 0.86 (SAD) and 0.79 (LAD). AI-based measurements were numerically stable across hardware configurations. **Conclusions**: Fully automated AI-based LN measurements in chest CT scans provide strong agreement with multi-reader consensus and high numerical stability. Automated measurement may support more standardized and reproducible oncologic imaging assessment.

## 1. Introduction

Precise, reproducible, and standardized measurement of imaging findings in computed tomography (CT) scans is crucial for accurate tumor staging and follow-up, directly influencing therapeutic decision-making and patient outcomes [1]. In oncologic imaging, measurement discrepancies may become clinically relevant when lymph node (LN) size is close to established staging or response thresholds, as small deviations around these cutoffs can potentially influence classification and subsequent management decisions. This underscores the need for robust and reproducible measurement techniques.

To ensure consistency and comparability, several staging and follow-up systems have been established over the course of the last few decades. The TNM system [1,2]—T = tumor, N = LN, and M = distant metastases—represents the reference for the classification of solid tumors, while the Lugano classification [3,4] is widely used for the classification of Hodgkin and non-Hodgkin lymphomas. For standardized therapeutic response assessment, Response Evaluation Criteria in Solid Tumors (RECIST) [5] and Response Evaluation Criteria in Lymphoma (RECIL) [6]/Lugano Criteria [3] are routinely applied in clinical practice.

In daily routine, these classification systems rely heavily on accurate lymph node (LN) size assessment, as nodal involvement is a key determinant of disease extent. For solid tumors [5], unidimensional measurements of LN short-axis diameters (SADs) are routinely performed during staging and restaging CT examinations, while for lymphomas [3,6], bidimensional measurements including both the SAD and long-axis diameter (LAD) are recommended. Consequently, precise and consistent LN measurements are critical, as different cancer entities apply specific size thresholds for nodal characterization. For example, in rectal cancer, mesorectal LNs ≥ 9 mm and lateral LNs ≥ 7 mm in long-axis diameter are considered suspicious, [7] while in RECIST 1.1, LNs ≥ 15 mm in short-axis diameter qualify as target lesions [5].

Despite their clinical importance, LN measurements are still predominantly performed manually. Manual or semi-automated segmentation and measurement are time-consuming, operator-dependent, and susceptible to considerable intra- and inter-reader variability, even among experienced radiologists [8,9]. Paradoxically, increasing the number of readers does not necessarily improve measurement consistency but may instead amplify variability, highlighting the limitations of subjective manual assessment in complex oncologic workflows.

Against this background, there has been growing interest in automated approaches for LN assessment in CT imaging. Previous studies have explored automatic LN detection [10,11,12,13], evaluated semi-automated measurement tools [8,14,15,16] and assessed inter-rater variability across institutions [8]. However, evidence regarding the feasibility and reliability of fully automated LN measurement systems remains limited, particularly in routine clinical settings.

In the context of artificial intelligence (AI)-based LN analysis, it is important to distinguish between detection, segmentation, and measurement tasks. Detection refers to the identification and location of an LN within an image. Segmentation involves delineating the precise boundaries of a detected node, typically by generating a voxel-wise mask. Measurement represents a subsequent step, in which quantitative metrics such as SAD and LAD are derived from the segmented structure according to established clinical guidelines. While detection and segmentation focus on identifying and outlining anatomical structures, accurate measurement is critical for staging and therapy response assessment, as it directly influences threshold-based clinical decision-making.

Fully automated AI-supported LN measurement techniques offer the potential to provide structured, observer-independent, and time-efficient evaluations, thereby improving measurement reproducibility and supporting standardized oncologic decision-making.

Despite the central role of lymph node size in oncologic staging and treatment monitoring, routine LN measurement remains a fundamentally subjective task and is prone to inter-reader variability. Although previous studies have explored LN detection algorithms and semi-automated measurement tools, robust evidence regarding the accuracy, reproducibility, and numerical stability of fully automated AI-based LN measurement workflows under routine clinical conditions remains limited. Moreover, validation against a large multi-reader consensus reference and assessment of hardware-related measurement stability have not been systematically investigated.

To our knowledge, this is the first study to comprehensively evaluate the accuracy, reproducibility, and hardware stability of a fully automated AI-based lymph node measurement workflow in contrast-enhanced chest CT using a large multi-reader consensus reference. By incorporating 13 radiologists, two independent reading rounds, and cross-platform validation, this study provides clinically relevant evidence for the robustness and real-world applicability of fully automated LN measurement.

The aim of this study was to address this gap by evaluating the precision and reproducibility of fully automated AI-based LN measurements (SAD and LAD) in contrast-enhanced chest CT scans and comparing them to measurements performed by multiple radiologists with varying levels of experience, which served as the ground truth (GT).

## 2. Materials and Methods

### 2.1. The Dataset Preparation

For this retrospective study, a total of 60 thoracic lymph nodes (LNs) were systematically pre-selected from contrast-enhanced chest CT scans of seven patients, based on predefined inclusion criteria to ensure coverage of different anatomical locations, size ranges, and imaging appearances. Patients were randomly selected from the institutional imaging archive between October 2016 and April 2020, after which eligible lymph nodes were identified within each examination, based on the availability of high-quality contrast-enhanced chest CT examinations acquired using standardized clinical protocols. To ensure representation of clinically relevant oncologic scenarios, patients referred for staging of histopathologically confirmed lung cancer (n = 4) and chronic lymphocytic leukemia (n = 3) were included, thereby covering both solid and hematologic malignancies.

All lung cancer examinations were performed on a 128-slice PET/CT system (Biograph mCT Flow 128 Edge, Siemens Healthineers, Erlangen, Germany). All chronic lymphocytic leukemia examinations were performed using a 256-slice CT system (iQon, Philips Medical Systems, Best, The Netherlands). Patients were scanned in the supine position in the cranio-caudal direction during inspiratory breath-hold. The routine clinical protocol included venous-phase imaging of the chest obtained after intravenous administration of 100 mL of non-ionic iodinated contrast medium (Accupaque 350 mg I/mL, GE Healthcare, Little Chalfont, UK) injected via an antecubital vein at a flow rate of 3–4 mL/s, with a scan delay of 50 s.

The following scan parameters were used: collimation, 128 × 64 × 0.625 mm; rotation time, 0.33 s; pitch, 0.671; tube voltage, 120 kVp; matrix size, 512 × 512. All axial images were reconstructed with a slice thickness of 2 mm.

**Figure 1:**
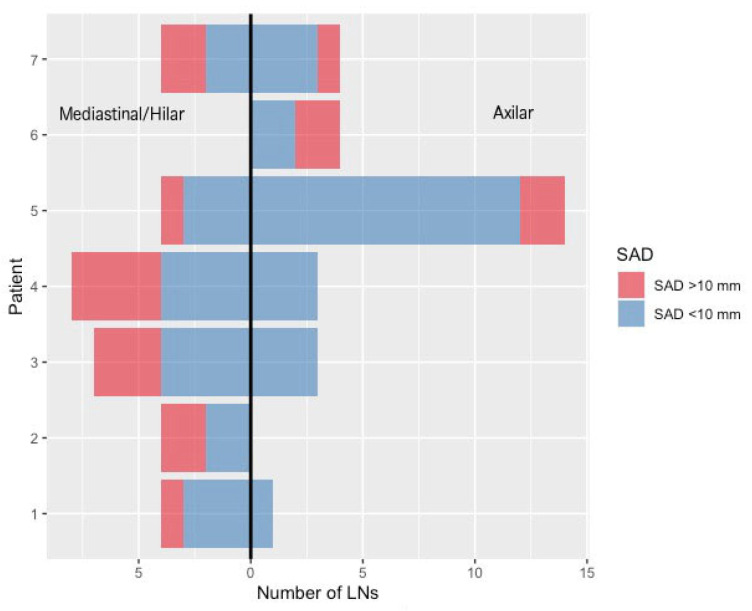
Included thoracic lymph nodes (LNs) per patient and anatomical region. The mirrored bar chart illustrates the number of mediastinal/hilar (**left**) and axillary (**right**) LNs included per patient. Bars are stratified by short-axis diameter (SAD), distinguishing LNs ≤ 10 mm and > 10mm, highlighting the inclusion of both normal-sized and enlarged nodes across patients and regions. LN = lymph node; SAD = short-axis diameter.

Lymph nodes were deliberately selected by an experienced radiologist (AII; 7 years of experience) according to predefined criteria to achieve a balanced distribution of mediastinal (n = 31) and axillary (n = 29) nodes, capturing variability in anatomical location, morphology, and size (Figure 1). Both normal-sized and pathological LNs were included, while extreme presentations such as bulky mediastinal or axillary disease were intentionally excluded to ensure robust and feasible measurement across all readers and methods. This selection strategy was designed to create a diverse yet controlled dataset, enabling reliable assessment of measurement precision and reproducibility across different readers and measurement modalities. The LN dataset included right- and left-sided axillary nodes (n = 14 and n = 15, respectively) and lymph nodes from multiple mediastinal stations and hilar regions (n = 31), as summarized in Table 1.

### 2.2. Dataset Evaluation

#### 2.2.1. Manual Assessment

A total of 13 radiologists with varying levels of experience (range: 2–10 years; mean: 5.5 years) manually measured the LAD and SAD of the 60 pre-selected LNs in the axial plane, in accordance with routine clinical practice. All manual measurements were performed using the institutional clinical PACS system (IMPAX EE, Agfa Healthcare, Mortsel, Belgium, Version R20 XIX). Each radiologist independently performed all measurements twice, with a minimum interval of four weeks between readings to minimize recall bias.

Selected LNs were pre-labeled and numbered (1–60) and were consistently referenced across both measurement sessions. Measurements from the first reading (run I) were denoted as SAD1 and LAD1, while measurements from the second reading (run II) were denoted as SAD2 and LAD2. All readers were blinded to clinical information, to measurements performed by other readers, and to their own prior measurements.

The readers included radiologists with 2 years (n = 2), 3 years (n = 1), 4 years (n = 2), 5 years (n = 2), 7 years (n = 4), 9 years (n = 1), and 10 years (n = 1) of experience, comprising seven resident physicians and six senior physicians. For subgroup analysis, readers were categorized into less-experienced (≤2 years) and more-experienced (>2 years) groups. As no universally accepted definition for reader experience categories exists in the literature, a pragmatic cutoff of ≤2 years versus >2 years of radiology experience was used to distinguish early-stage trainees from more experienced readers.

**Figure 2:**
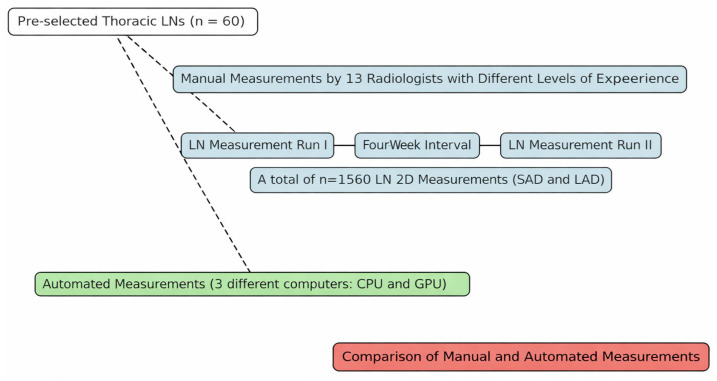
Overview of the data evaluation workflow. Sixty pre-selected thoracic lymph nodes were evaluated using manual multi-reader measurements and a fully automated AI-based approach. Thirteen radiologists performed two independent measurement rounds separated by a four-week interval. Automated measurements were repeated on three different computer systems using both CPU and GPU execution. Manual and AI-based measurements were subsequently compared. LN = lymph node; LAD = long-axis diameter; SAD = short-axis diameter; CPU = central processing unit; GPU = graphics processing unit.

An overview of the data evaluation workflow is provided in Figure 2.

#### 2.2.2. Ground Truth

To establish a robust and clinically meaningful ground truth (GT), the median of all measurements obtained from the 13 readers for each individual LN was used for further analysis. Given the absence of an objective anatomical reference standard for LN diameter in routine clinical CT imaging and the inherent variability of manual measurements, no single reader measurement can be considered definitively correct. The median was therefore selected as a consensus-based estimate that reflects routine clinical measurement behavior while reducing the influence of potential outliers. In this context, multi-reader consensus approaches represent a pragmatic reference standard for measurement studies and approximate real-world clinical decision-making, where lymph node assessment relies on standardized radiological interpretation rather than a single authoritative reference. However, such consensus-based references may still be influenced by systematic measurement biases shared among readers.

#### 2.2.3. AI-Based Automatic Assessment

The automatic measurement of LNs in this study was based on a fully automated multi-stage workflow using an existing vendor-developed research tool. First, LN segmentation was performed using a previously validated convolutional neural network (CNN) integrated as a research prototype within the IntelliSpace Discovery platform (Version 3.0, Philips Medical Systems, Best, The Netherlands). The CNN used for LN segmentation was a previously published 3D fully CNN designed for automated thoracic LN detection and segmentation [11,12]. The model was trained on annotated CT datasets using supervised learning and optimized using standard cross-entropy-based loss functions. Detailed architectural configuration, training parameters, and hyperparameter settings are described in the original publication. In the present study, the model was applied as a fixed, pre-trained network without further modification or fine-tuning.

Second, connected component analysis was applied to the resulting segmentation masks to identify individual lymph nodes. Third, the SAD and LAD were automatically derived using the Multi-Modality Tumor Tracking application available in the IntelliSpace Portal (Version 12.1, Philips Healthcare, Amsterdam, The Netherlands), following established oncologic measurement guidelines [4,5].

To ensure that hardware-related effects such as rounding and numerical implementation differences did not influence the results, AI-based segmentation and automated SAD/LAD computation were repeated on three independent computer systems, with each analysis performed on both the central processing unit (CPU) and the graphics processing unit (GPU). Hardware specifications are summarized in Table 2.

The purpose of this study was not to develop, train, or optimize an AI model but to evaluate the measurement stability and reproducibility of a fully automated AI-driven workflow in comparison to routine manual measurements. Accordingly, the CNN was used as a fixed and previously validated segmentation component, and no model training, fine-tuning, or performance optimization was performed within the scope of this study. This design reflects a real-world clinical scenario, in which AI systems are applied as standardized measurement tools rather than continuously adapted algorithms, enabling direct comparison between automated and human-derived measurements under routine conditions.

### 2.3. Statistical Analysis

Statistical analysis was performed using Python (version 3.7) with the libraries NumPy, pandas, SciPy, and scikit-posthocs. SAD and LAD measurements obtained from the AI-based workflow and from the two measurement runs performed by each reader were analyzed for all 60 lymph nodes. All analyses were conducted at the individual LN level, without aggregation across multiple nodes. Given the non-normal distribution of the measurement data, Friedman chi-square tests for paired samples were used to assess overall differences in SAD and LAD measurements across all readers and the AI-based method for each individual LN. When the Friedman test indicated statistical significance, pairwise comparisons between individual readers and the AI-based measurements, as well as between reader pairs, were performed using Conover post hoc tests. *p*-values were adjusted using the Holm–Bonferroni method to control for multiple testing.

To assess agreement and reproducibility, the intraclass correlation coefficient (ICC) and the concordance correlation coefficient (CCC) were calculated. The ICC was used to quantify inter- and intra-reader reliability across repeated measurements, while the CCC was applied to evaluate agreement between AI-based measurements and the ground truth (GT) by jointly assessing precision and accuracy. For each individual LN, the median of both measurement runs across all 13 readers served as the GT. Statistical significance was defined as a two-sided *p*-value < 0.01 to reduce the risk of a Type I error in the context of multiple comparisons.

## 3. Results

### 3.1. Manual Measurements

In total, 1560 manual measurements were analyzed. Manual LAD measurements across the 60 LNs ranged from 4 to 39 mm, while manual SAD measurements ranged from 3 to 26 mm, reflecting the natural spectrum of normal-sized and enlarged LNs within the selected cohort. An overview of LAD and SAD values obtained during measurement runs I and II is provided in Table 3.

Inter-reader variability was assessed using the Friedman chi-square test, which revealed statistically significant differences between readers (*p* < 0.01), with subsequent Conover post hoc testing applied for pairwise comparison. These findings indicate relevant variability across manual measurements, which was further explored in the post hoc analysis.

#### Manual Measurements of the Individual Readers

**Figure 3:**
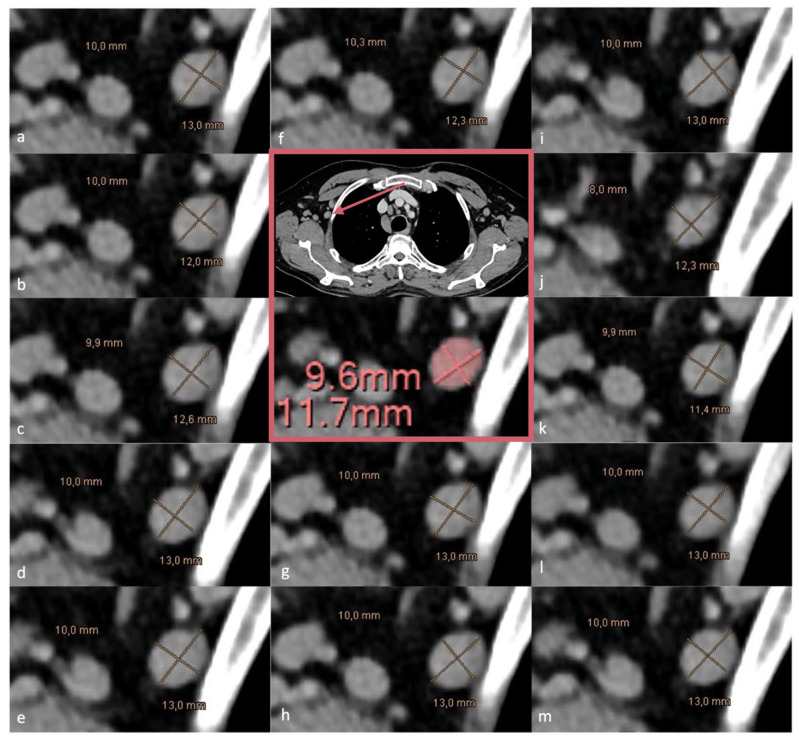
Example of reading run I (SAD and LAD) of one of the axillary LNs showing the difference between the individual readers (**a**–**m**) and the measurement generated by the AI (pink box). Legend: AI—artificial intelligence. LN = lymph node; LAD = long-axis diameter; SAD = short-axis diameter.

From the 78 possible reader pair combinations (13 × 12/2), statistically significant differences were observed in 16 comparisons for SAD run I, 31 for SAD run II, 18 for LAD run I, and 31 for LAD run II (Supplementary Material, Table S1). For example, one reader measured a given axillary lymph node as 8 × 12 mm, whereas another reader measured the same lymph node as 10 × 13 mm. Figure 3 illustrates an exemplary axillary LN measurement performed by all 13 radiologists in comparison to the AI-based approach.

To further characterize these differences, concordance correlation coefficients (CCCs) were calculated, yielding values ranging from 0.85 to 0.99. In most reader comparisons, CCC values indicated excellent agreement (>0.90); however, several comparisons demonstrated only good agreement (0.85–0.90). These findings indicate that, although statistically significant differences between readers were present, the absolute measurement deviations were generally small.

No statistically significant intra-reader differences were observed between measurement runs I and II for any reader (*p* > 0.01). Correspondingly, CCC values between run I and run II were high for all readers when compared to the GT, with values of 0.98 for LAD and 0.97 for SAD, confirming excellent intra-reader reliability (Table 4).

### 3.2. AI-Based Measurements

To confirm the consistency and numerical stability of the automated measurements, AI-based LN segmentation and SAD/LAD computation were performed on three independent computer systems, with each analysis executed on both the GPU and CPU. Each configuration was repeated twice, resulting in 12 independent runs and a total of 720 AI-based measurements. No measurable differences in SAD or LAD values were observed across machines, processing units, or repeated runs.

Minor variations in the underlying probability maps were observed (maximum deviation ≤ 0.004); however, these differences disappeared after thresholding and had no impact on the final LN measurements.

### 3.3. Manual vs. AI-Based Measurements

#### 3.3.1. Median of the Readers (GT) vs. AI-Based Measurements

When comparing AI-based measurements to the GT, no statistically significant differences were observed for either SAD or LAD across both measurement runs (all *p* > 0.01). Similarly, no significant differences were found between AI-based measurements and the median values of experienced readers, nor between AI-based measurements and the median values of less-experienced readers (all *p* = 1.0), for both SAD and LAD.

#### 3.3.2. Individual Readers vs. AI-Based Measurements

When comparing AI-based measurements with those of individual readers, the Friedman chi-square test demonstrated statistically significant differences (*p* < 0.01). Subsequent Conover post hoc testing revealed significant pairwise differences between the AI-based measurements and a limited number of individual readers.

**Figure 4:**
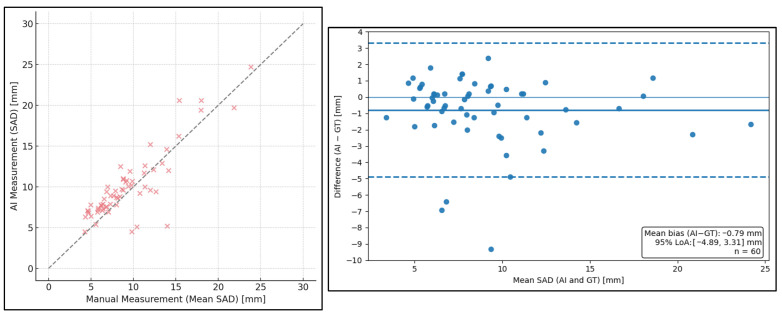
Agreement between AI-based and ground truth short-axis diameter (SAD) measurements. (**left**) Scatter plot showing AI-based SAD measurements versus the ground truth (GT), defined as the median of all 13 reader measurements. The solid diagonal line represents the line of identity, indicating perfect agreement between AI-based and manual measurements. (**right**) Bland–Altman plot illustrating the difference between AI-based and GT SAD measurements as a function of their mean. The solid bold horizontal line represents the mean measurement bias, while the dashed lines indicate the 95% limits of agreement. AI = artificial intelligence; SAD = short-axis diameter; GT = ground truth.

Specifically, significant differences were observed between the AI-based measurements and two readers for SAD run I, four readers for SAD run II, one reader for LAD run I, and two readers for LAD run II (Figure 4). These findings indicate that deviations occurred only in a subset of readers rather than systematically across all readers, while overall agreement between AI-based measurements and the multi-reader GT remained high.

## 4. Discussion

To ensure the accuracy and reproducibility of quantitative data derived from CT scans in oncologic patients, various staging and follow-up guidelines have been developed over the years. Despite these standardized frameworks, image evaluation in routine clinical practice is still predominantly performed manually by radiologists, which is time-consuming and inherently affected by inter- and intra-reader variability [8,9].

Since accurate assessment of metastatic nodal extent at initial staging and during therapy monitoring is crucial for optimized treatment planning and individual patient outcomes, precise and reproducible LN measurement remains a key requirement in oncologic imaging. AI-based measurement techniques offer the potential to address these challenges by providing standardized and observer-independent assessments, thereby improving the consistency and efficiency of cancer staging and treatment decisions.

Previous studies have investigated automatic LN detection in CT images [10,11,12,13] and the benefits of semi-automated LN measurement tools compared to manual measurements [8,14,15,16]. However, evidence regarding the feasibility and accuracy of fully automated LN measurement workflows remains limited. Accordingly, the aim of this study was to evaluate the accuracy and reproducibility of fully automated AI-based LN measurements of SAD and LAD in staging chest CT scans, while LN detection performance itself was beyond the scope of this work. However, reliable automated detection represents a critical prerequisite for the implementation of fully automated measurement workflows in clinical practice.

Buerke et al. [16] evaluated the accuracy and reproducibility of semi-automated LN assessment in 742 cervical, thoracic, and abdominal LNs. Fully correct semi-automated segmentation without manual correction was achievable in only 64.7% of cases, although intraclass correlation analysis demonstrated good agreement between manual and semi-automated measurements (r = 0.70–0.81). These findings highlight both the potential and the limitations of semi-automated approaches, particularly regarding their dependence on user interaction.

Previous studies investigating AI-based or semi-automated LN analysis have faced several limitations. Many focused on detection alone without precise size quantification [10,11,12,13] or relied on semi-automated tools requiring user input or manual correction [8,14,15,16], thereby introducing observer-dependent variability. In addition, manual refinement of segmentations, as reported in some studies [15,16], may have produced biased reproducibility assessments. Heterogeneous datasets spanning multiple anatomical regions further complicated measurement standardization, particularly for small or anatomically complex LNs.

In contrast, this study evaluates a fully automated AI-based system for LN segmentation and measurement without human interaction or correction. By focusing on a well-defined dataset of thoracic LNs from two oncologic entities (lung cancer and CLL) and incorporating 13 radiologists across two independent reading rounds, this work provides a standardized and clinically relevant assessment of measurement accuracy and reproducibility. The combination of full automation and a large number of expert-derived measurements (n = 2280) addresses key limitations of prior studies and supports clinical feasibility.

Overall, AI-based LN measurements demonstrated high accuracy and concordance with manual assessments, showing no significant bias relative to the GT. High CCC values confirmed good agreement between AI-based measurements and the GT, with values ranging from approximately 0.79 to 0.86. In contrast, intra-reader agreement between repeated manual measurements was higher, with CCC values around 0.97–0.99, reflecting excellent measurement consistency for individual readers.

In contrast, significant differences were observed between individual readers for both SAD and LAD measurements, reflecting inter-reader variability and limited reproducibility of manual measurements in routine clinical practice. These findings are consistent with previous studies comparing manual and semi-automated LN measurements [8,15,16], which likewise reported greater variability in manual assessments. To the best of our knowledge, this study is the first to systematically evaluate the feasibility and reproducibility of fully automated LN measurements in chest CT scans, representing a meaningful contribution to the existing literature.

In our dataset, AI-based measurements showed no significant deviation from the median of radiologist assessments, and no consistent pattern of under- or overestimation was observed. However, factors such as small LN size, irregular shape, motion artifacts, or low contrast at anatomical borders can impact segmentation accuracy in clinical practice. LNs adjacent to vascular structures or located in regions with high anatomical complexity (e.g., the hilum) may be particularly challenging for AI algorithms. Although our dataset did not reveal such effects, these remain important considerations for broader clinical deployment. Moreover, the results show no statistically significant difference between the measurements of the experienced and non-experienced readers. Since the factor “reading time” was not recorded for the LN measurements, a “compensatory effort” by the inexperienced readers cannot be ruled out.

Comparison with the existing literature further supports these findings. Previous studies on semi-automated LN analysis reported inter-reader agreement levels with ICC values ranging from 0.70 to 0.81 [8,16]. In contrast, the fully automated approach evaluated here achieved higher agreement, with CCC values between 0.97 and 0.99 across reader groups, without requiring any manual interaction, underscoring the potential of full automation to deliver reproducible measurements comparable to expert consensus.

Although direct timing data were not collected in this study, prior research in thoracic imaging has demonstrated that AI-based tools can improve workflow efficiency. For example, Kurmukov et al. [18] reported a reduction in average chest CT reading time of approximately 20.6% with AI assistance. While these findings relate to full chest CT interpretation and not specifically to LN measurement, they support the broader potential of AI to streamline radiological workflows. In clinical practice, fully automated LN measurement could support several integration scenarios. First, automated pre-generation of LN measurements during image preprocessing could provide radiologists with preliminary measurements for staging and follow-up examinations. Second, AI-derived measurements could function as a “second reader” or pre-read system, supporting, but not replacing, the final radiologist interpretation. Third, standardized automated measurements may facilitate multi-center studies and clinical trials by reducing inter-reader variability and minimizing the need for extensive manual multi-reader annotations.

However, fully automated measurement of LNs remains technically challenging. LNs are often small, show variable morphology, and may exhibit similar attenuation to adjacent vascular or mediastinal structures, which can complicate reliable segmentation. In addition, partial volume effects, motion artifacts, and anatomical complexity (particularly in the hilar and mediastinal regions) may impair accurate boundary delineation. The application of standardized diameter-based criteria further requires consistent orientation and precise border detection, highlighting that fully automated LN measurement workflows remain an area of ongoing methodological development.

This study has several limitations. Although a large number of measurements were analyzed (n = 2280), the dataset comprised 60 LNs from seven patients, which may limit patient-level generalizability. The present work was designed as a technical validation study focusing on measurement reproducibility rather than population-based diagnostic performance. Furthermore, multiple LNs from the same patient were analyzed as individual observations, and potential intra-patient clustering effects were not explicitly modeled. Future studies with larger, multi-center cohorts and hierarchical statistical approaches could further strengthen generalizability.

Another limitation relates to the deliberate exclusion of extreme nodal presentations such as bulky conglomerate disease. This decision was made to ensure standardized and comparable diameter measurements across readers, as measurement conventions in confluent nodal masses may vary substantially. While both normal-sized and enlarged LNs from different thoracic regions were included, performance in highly complex or mass-like nodal disease requires further investigation.

From an artificial intelligence perspective, additional limitations should be acknowledged. The CNN was applied as a fixed, pre-trained model without external multi-center validation in this study, and potential domain shift effects related to scanner types, acquisition protocols, and patient populations were not specifically assessed. In addition, the influence of image quality and artifacts on segmentation and measurement performance was not systematically evaluated. Although the dataset consisted of contrast-enhanced CT examinations with relatively consistent imaging conditions, factors such as motion artifacts, variable contrast enhancement, metal implants, and differences in patient body habitus may affect automated segmentation and measurement reliability in clinical practice. Future studies should therefore evaluate the robustness of the proposed AI workflow across more heterogeneous imaging environments.

## 5. Conclusions

Fully automated AI-based lymph node measurements in chest CT scans demonstrated high reproducibility and strong agreement with manual radiologist assessments. These findings highlight the potential of AI to support standardized and consistent LN evaluation in oncologic imaging, where measurement reliability is critical for staging and therapy monitoring.

Although further prospective and multi-center validation is warranted, the AI-based approach may enhance measurement consistency and efficiency in routine clinical workflows, supporting radiologists during cancer staging, follow-up, and treatment response assessment.

## Figures and Tables

**Table 1:** Location of the included LNs and overview of the number of LNs/location. LN levels are in accordance with the Mountain–Dresler modification of the American Thoracic Society LN classification [17]. L = left; R = right; LN = lymph node.

LN Location	Number of Included LNs
axillar L	15
axillar R	14
hilar L	1
hilar R	4
level 2 L	3
level 2 R	2
level 3a	1
level 4 L	5
level 4 R	9
level 5	3
level 7	2
level 8	1
**Total**	**60**

**Table 2:** Details on the CPU, GPU and RAM of the three computers (Comp. 1–3) used for the network inference for the automatic measurements. CPU—central processing unit; GPU—graphics processing unit.

	CPU	GPU	RAM
Comp. 1	Intel^®^ Xeon^®^ CPU E5-2603 v4 @1.70 GHz	NVIDIA GeForce RTX 1080 Ti	64 GB
Comp. 2	Intel^®^ Xeon^®^ Gold 5118 CPU @2.30 GHz	NVIDIA GeForce RTX 2080 Ti	512 GB
Comp. 3	Intel^®^ Xeon^®^ Silver 4210R CPU @2.40 GHz	NVIDIA GeForce RTX 3080	512 GB

**Table 3:** Overview of all LN measurements (mm). Legend: min.—smallest LAD/SAD; max.—largest LAD/SAD. SAD = short-axis diameter; LA = long-axis diameter; std. dev. = standard deviation; average—average for all 13 readers.

	Manual Assessment	
	Reading Run I	Reading Run II
LAD		
min.	4.00	4.50
max.	38.00	39.00
average	13.60	13.61
std. dev.	6.44	6.46
SAD		
min.	3.40	3.00
max.	26.00	26.00
average	9.39	9.37
std. dev.	4.24	4.29

**Table 4:** Concordance correlation coefficients (CCCs) and interclass correlation coefficients (ICCs) between the AI-based measurements in reading run I and II and the median of all readers as the ground truth (GT). A high level of concordance between the AI-based measurements and the median of all readers, as well as the median of the expert and non-experienced readers was shown for all measurements. SAD = short-axis diameter; LAD = long-axis diameter; AI = artificial intelligence; CCC = concordance correlation coefficient; ICC = interclass correlation coefficient.

	CCC		ICC	
Reading run I	SAD AI	LAD AI	SAD AI	LAD AI
Median all readers	0.86	0.79	0.86	0.79
Median expert readers	0.86	0.78	0.86	0.79
Median non-experienced readers	0.86	0.80	0.84	0.79
Reading run II	SAD AI	LAD AI	SAD AI	LAD AI
Median all readers	0.85	0.79	0.85	0.79
Median expert readers	0.85	0.80	0.86	0.80
Median non-experienced readers	0.86	0.80	0.85	0.80

## Data Availability

The dataset is available upon request to the corresponding author: andra.iuga@uk-koeln.de.

## Supplementary Materials

The following supporting information can be downloaded at: https://www.mdpi.com/article/10.3390/diagnostics16070967/s1, Table S1: Comparison of the individual SAD measurements (run I) for all readers; Table S2: Comparison of previous studies on lymph node assessment in CT imaging and the present study.

## Abbreviations

The following abbreviations are used in this manuscript:

AI	artificial intelligence
CCC	concordance correlation coefficient
CLL	chronic lymphocytic leukemia
CNN	convolutional neural network
CPU	central processing unit
CT	computed tomography
GT	ground truth
GPU	graphics processing unit
ICC	intraclass correlation coefficient
LAD	long-axis diameter
LN	lymph node
RECIL	Response Evaluation Criteria in Lymphoma
RECIST	Response Evaluation Criteria in Solid Tumors
SAD	short-axis diameter
TNM	tumor–node–metastasis
