# Supplementary material for: Fully Automated AI-Based Lymph Node Measurements in Chest CT: Accuracy and Reproducibility Compared with Multi-Reader Assessment
Source: Diagnostics (Basel). 2026 Mar 24;16(7):967. doi: 10.3390/diagnostics16070967 (PMC13072389; doi:10.3390/diagnostics16070967)
Supplement: Supplementary file 1 [file diagnostics-16-00967-s001.zip › diagnostics-4146877-supplementary.pdf]

Supplementary Material

Table S1. Comparison of the individual SAD measurements (run I) for all readers.

	reader 1	reader2	reader 3	reader 4	reader 5	reader 6	reader 7	reader 8	reader 9	reader 10	reader 11	reader 12	reader 13
reader 1	1.000												
reader2	1.000	1.000											
reader 3	1.000	1.000	1.000										
reader 4	0.016	0.304	1.000	1.000									
reader 5	< 0.01	< 0.01	0.403	1.000	1.000								
reader 6	1.000	1.000	1.000	0.150	< 0.01	1.000							
reader 7	1.000	1.000	1.000	1.000	0.467	1.000	1.000						
reader 8	0.289	1.000	1.000	1.000	1.000	1.000	1.000	1.000					
reader 9	1.000	1.000	1.000	1.000	0.033	1.000	1.000	1.000	1.000				
reader 10	1.000	1.000	1.000	0.011	< 0.01	1.000	1.000	0.215	1.000	1.000			
reader 11	1.000	1.000	0.147	< 0.01	< 0.01	1.000	0.125	< 0.01	1.000	1.000	1.000		
reader 12	1.000	1.000	1.000	< 0.01	< 0.01	1.000	1.000	0.033	1.000	1.000	1.000	1.000	
reader 13	< 0.01	< 0.01	< 0.01	1.000	1.000	< 0.01	< 0.01	0.866	< 0.01	< 0.01	< 0.01	< 0.01	1.000

When considering the readers individually, significant inter-reader differences were shown between several readers both for LAD and SAD for both measurements. For example, SAD measurements were significantly different in 18 out of 78 comparisons for the 1st measurement (run I). Significant differences are marked in red.

Table S2. Comparison of previous studies on lymph node assessment in CT imaging and the present study.

Study	Year	Approach	Fully Automated	Manual Interaction Required	No. of LNs	Reference Standard	Main Focus
Buerke et al.[16]	2010	Semi-automated segmentation	No	Yes	742	Manual measurement	Accuracy & reproducibility
Weßling et al.[15]	2012	Semi-automated LN analysis	No	Yes		Manual measurement	Therapy response classification
Höink et al.[8]	2014	Semi-automated measurement	No	Yes		Manual measurement	Multi-center comparison
Iuga et al.[13]	2021	AI-based LN detection	Yes (segmentation)	No		Manual annotation	Detection & segmentation

Study	Year	Approach	Fully Automated	Manual Interaction Required	No. of LNs	Reference Standard	Main Focus
**Present study**	2026	Fully automated segmentation + measurement	**Yes**	**No**	60 (2,280 measurements)	Median of 13 radiologists (2 rounds)	Accuracy, reproducibility & hardware stability
